# Clinical significance of cigarette smoking and dust exposure in pulmonary alveolar proteinosis: a Korean national survey

**DOI:** 10.1186/s12890-017-0493-4

**Published:** 2017-11-21

**Authors:** Ji An Hwang, Joo Han Song, Jung Hoon Kim, Man Pyo Chung, Dong Soon Kim, Jin Woo Song, Young Whan Kim, Sun Mi Choi, Seung Ick Cha, Soo Taek Uh, Choon-Sik Park, Sung Hwan Jeong, Yong Bum Park, Hong Lyeol Lee, Jong Wook Shin, Eun Joo Lee, Yangjin Jegal, Hyun Kyung Lee, Jong Sun Park, Moo Suk Park

**Affiliations:** 1 Department of Pulmonary and Critical Care Medicine, University of Ulsan College of Medicine, Asan Medical Center, Seoul, South Korea; 2 Division of Pulmonology, Department of Internal Medicine, Institute of Chest Diseases, Severance Hospital, Yonsei University, College of Medicine, Seoul, South Korea; 3 Division of Pulmonary and Critical Care Medicine, Samsung Medical Center, Sungkyunkwan University School of Medicine, Seoul, South Korea; 4 Division of Pulmonary and Critical Care Medicine, University of Ulsan College of Medicine, Asan Medical Center, Seoul, South Korea; 5 Division of Pulmonary and Critical Care Medicine, Department of Internal Medicine and Lung Institute, Seoul National University College of Medicine, Seoul, South Korea; 6 Division of Pulmonary and Critical Care Medicine, Department of Internal Medicine, Kyungpook National University Hospital, Daegu, South Korea; 7 Division of Allergy and Respiratory Medicine, Department of Internal Medicine, Soonchunhyang University Seoul Hospital, Seoul, South Korea; 8 Division of Allergy and Respiratory Medicine, Department of Internal Medicine, Soonchunhyang University Bucheon Hospital, Bucheon, South Korea; 9 Division of Pulmonology, Department of Internal Medicine, Gachon University Gil Medical Center, Incheon, South Korea; 10 Division of Pulmonary, Allergy and Critical Care Medicine, Department of Internal Medicine, Hallym University Kangdong Sacred Heart Hospital, Seoul, South Korea; 11 Pulmonary Division, Department of Internal Medicine, Inha University Hospital, Incheon, South Korea; 12 Division of Pulmonary Medicine, Department of Internal Medicine, Chung Ang University College of Medicine, Seoul, South Korea; 13 Division of Respiratory and Critical Care Medicine, Department of Internal Medicine, Korea University Anam Hospital, Korea University College of Medicine, Seoul, South Korea; 14 Division of Pulmonary Medicine, Department of Internal Medicine, Ulsan University Hospital, University of Ulsan College of Medicine, Ulsan, South Korea; 15 Division of Critical Care and Pulmonary Medicine, Department of Internal Medicine, Inje University Busan Paik Hospital, Busan, South Korea; 16 Division of Pulmonary and Critical Care Medicine, Department of Internal Medicine, Seoul National University College of Medicine, Seoul National University Bundang Hospital, Seongnam, South Korea; 17 Division of Pulmonology, Department of Internal Medicine, Institute of Chest Diseases, Severance Hospital, Yonsei University, College of Medicine, 50-1 Yonsei-ro, Seodaemun-gu, Seoul, 120-752 South Korea

**Keywords:** Disease severity, Dust exposure, Pulmonary alveolar proteinosis, Smoking

## Abstract

**Background:**

This study aimed to investigate clinical characteristics of Korean PAP patients and to examine the potential risk factors of PAP.

**Methods:**

We retrospectively reviewed medical records of 78 Korean PAP patients diagnosed between 1993 and 2014. Patients were classified into two groups according to the presence/absence of treatment (lavage). Clinical and laboratory features were compared between the two groups.

**Results:**

Of the total 78 PAP patients, 60% were male and median age at diagnosis was 47.5 years. Fifty three percent were ever smokers (median 22 pack-years) and 48% had a history of dust exposure (metal 26.5%, stone or sand 20.6%, chemical or paint 17.7%, farming dust 14.7%, diesel 14.7%, textile 2.9%, and wood 2.9%). A history of cigarette smoking or dust exposure was present in 70.5% of the total PAP patients, with 23% having both of them. Patients who underwent lavage (*n* = 38) presented symptoms more frequently (38/38 [100%] vs. 24/40 [60%], *P* < 0.001) and had significantly lower PaO_2_ and DL_CO_ with higher D(A-a)O_2_ at the onset of disease than those without lavage (*n* = 40) (*P* = 0.006, *P* < 0.001, and *P* = 0.036, respectively). Correspondingly, the distribution of disease severity score (DSS) differed significantly between the two groups (*P* = 0.001). Based on these, when the total patients were categorized according to DSS (low DSS [DSS 1–2] vs. high DSS [DSS 3–5]), smoking status differed significantly between the two groups with the proportion of current smokers significantly higher in the high DSS group (11/22 [50%] vs. 7/39 [17.9%], *P =* 0.008). Furthermore, current smokers had meaningfully higher DSS and serum CEA levels than non-current smokers (*P* = 0.011 and *P* = 0.031), whereas no difference was found between smokers and non-smokers. Regarding type of exposed dust, farming dust was significantly associated with more severe form of PAP (*P* = 0.004).

**Conclusion:**

A considerable proportion of PAP patients had a history of cigarette smoking and/or dust exposure, suggestive of their possible roles in the development of PAP. Active cigarette smoking at the onset of PAP is associated with the severity of PAP.

## Background

Pulmonary alveolar proteinosis (PAP) is a rare disease characterized by the accumulation of excessive surfactant lipids and proteins within alveoli, leading to the impairment of gas exchange [1–3]. Clinical manifestations vary from no symptoms to progressive respiratory failure [3, 4]. Granulocyte-macrophage colony-stimulating factor (GM-CSF) deficiencies or defects in GM-CSF receptors are known to be related to the pathogenesis. GM-CSF is required for the terminal differentiation of alveolar macrophages (AMs) which play a key role in the clearance of normal surfactant proteins and phospholipids. Three different forms of PAP have been identified: idiopathic (primary or autoimmune), secondary, and hereditary [3–6]. More than 90% of PAP patients have idiopathic PAP (iPAP), a primary acquired disorder without familial predisposition in which GM-CSF neutralizing auto-antibodies are present [4, 7]. Secondary PAP is associated with various underlying diseases (hematologic malignancies, immunodeficiency disorders, infections) or inhalation injuries that cause AM dysfunction or deficiency in AM number [4, 8]. Hereditary PAP results from homozygous mutations of the genes encoding surfactant proteins and the ABCA3 transporter or from defects of the GM-CSF receptor [9].

To date, there have been several cross-sectional or retrospective studies enrolling a large cohort of PAP patients [10–13]. Although the studies have shown similar results in the distribution of age, male predominance, clinical manifestations and treatment methods, there have been demonstrated differences in suspected risk factors of PAP such as smoking and dust exposure.

Therefore, our present study aimed to investigate clinical characteristics of Korean PAP patients and to examine the potential risk factors of PAP with a focus on smoking and dust exposure.

## Methods

### Study participants

The Korean Interstitial Lung Disease Study Group retrospectively reviewed the medical records of PAP patients diagnosed between 1993 and 2014. The data were collected from 15 tertiary referral hospitals as a national multi-center survey.

This study was approved by the Institutional Review Board (IRB) of Severance Hospital (IRB approval number: 4–2009-0372). As this was a retrospective study, the IRB waived the requirement for informed consent.

### Diagnosis

The diagnosis of PAP was based on diagnostic bronchoalveolar lavage (BAL) finding [14, 15], characteristic radiologic findings, and/or histopathologic findings of specimens obtained by surgical lung biopsy or transbronchial biopsy (TBB) and/or cytologic findings in BAL samples. Characteristic radiologic findings showed interlobular septal thickening in multiple lobes and/or diffuse, patchy, geographic appearance of ground glass opacities [16]. Diagnostic histopathologic findings included intraalveolar eosinophilic, periodic acid-Schiff (PAS)-positive material, intracellular surfactant inclusion bodies in AMs, and turbid, PAS-positive, eosinophilic BAL fluid [14, 15].

### Data collection

Baseline characteristics of the patients including age, sex, smoking status, and the presence/absence of symptoms at initial disease onset were investigated based on the medical chart review, while data on a history of dust exposure were obtained through a detailed survey of an occupational history in patients with PAP. Patients were classified into 3 categories based on the smoking status in Tables 3 and 4 (never smokers, ex-smokers, and current smokers). Additional analyses were conducted according to the presence/absence of current active smoking at the time of PAP diagnosis (Fig. 3a; current smokers vs. non-current smokers) or smoking history itself including past and current smoking (Fig. 3b; smokers vs. non-smokers). ‘Non-current smokers’ included both never-smokers and ex-smokers as contrasted with ‘current smokers’ and ‘smokers’ included both ex-smokers and current smokers. The extent and the pattern of radiologic lung involvements, blood gas analyses, pulmonary function test (PFT) results including diffusing capacity of the lung for carbon monoxide (DL_CO_) and forced vital capacity (FVC), and the levels of known serum biomarkers including lactate dehydrogenase (LDH) [5, 10, 12, 17–21] and carcinoembryonic antigen (CEA) [10, 12, 20, 22–25] at the time of diagnosis were also investigated. We attained additional data on blood gas analyses and PFT results following therapeutic lavage where possible.

### Assessment of disease severity score

Each patient was assigned a PAP disease severity score (DSS) based on the presence/absence of symptoms and the degree of PaO_2_ at initial diagnosis, as previously described [25]. The categories of score ranged from DSS 1 to DSS 5: DSS 1 = no symptoms and PaO_2_ ≥ 70 mmHg, DSS 2 = symptomatic and PaO_2_ ≥ 70 mmHg, DSS 3 = 60 mmHg ≤ PaO_2_ < 70 mmHg, DSS 4 = 50 mmHg ≤ PaO_2_ < 60 mmHg, DSS 5 = PaO_2_ < 50 mmHg.

### Statistics

Parametric data were presented as mean (± standard deviation) and nonparametric data as median and interquartile range (IQR). Categorical variables were expressed as either percentage of the total or number as appropriate. Numeric data were compared using Student’s t-test or Mann-Whitney U-test, and categorical variables were compared using the Chi-square or Fisher’s exact test. The degree of correlation between variables was evaluated using Pearson’s or Spearman’s correlation coefficient.

## Results

**Fig. 1:**
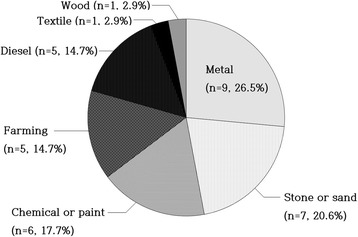
The types and the composition of exposed dust in patients with a history of dust exposure (*n* = 34)

Baseline characteristics are summarized in Table 1. A total of 78 patients from multiple centers in Korea were enrolled including 75 patients with iPAP and 3 patients with secondary PAP with underlying diseases such as lung cancer, lymphoma, and pulmonary tuberculosis. The median age at diagnosis was 47.5 years. Forty-seven (60%) patients were male and 39 (53%) patients were ever smokers. Sixty-two (80%) patients were symptomatic at diagnosis of PAP. The proportion of patients with low DSS (DSS 1–2) was higher compared with that of patients with high DSS (DSS 4–5) (65% vs. 11%). Thirty-four (48%) patients had a history of dust exposure. The types and the composition of exposed dust are presented as Fig. 1. A history of cigarette smoking or dust exposure was present in 55 (71%) patients with PAP, with 18 (23%) patients having both of them. Thirty-two (41%) patients exhibited diffuse bilateral lung involvements and 32 (64%) of the evaluable 50 patients typical crazy paving appearance in their radiologic findings. 35 (45%) patients were diagnosed through surgical lung biopsy while 43 (55%) patients through TBB with BAL alone (Table 2). Regarding treatment, 38 (49%) patients were treated with whole or segmental lung lavage whereas 30 (50%) patients were closely observed without active treatment. Most patients survived except for 3 patients with the causes of death including respiratory failure, hepatocellular carcinoma, and pneumonia. When a significant progression of respiratory symptoms with a subsequent application of additional therapeutic lavage was considered recurrence, PAP recurred in 11 (14%) of the total 78 patients during the follow-up period.

**Table 1:** Baseline Characteristics of the patients diagnosed with PAP between 1993 and 2014 in Korea (*n* = 78)

Variables^a^	*N* = 78
Classification	
Idiopathic	75 (96.2)
Secondary	3 (3.8)
Age (median years [IQR])	47.5 (42.5–59)
Male	47 (60.3)
Smoking status (*n* = 73)	
Never-smoker	34 (46.6)
Ex-smoker	18 (24.6)
Current smoker	21 (28.8)
Symptoms	
Symptomatic	62 (79.5)
Asymptomatic	16 (20.5)
Disease severity score^b^ (*n* = 65)	
1	9 (13.8)
2	33 (50.8)
3	16 (24.6)
4	4 (6.2)
5	3 (4.6)
Presence of dust exposure (*n* = 71)	34 (47.9)
Extent of radiologic involvement	
Diffuse bilateral lung involvement	32 (41.0)
Chest CT pattern (*n* = 50)	
Ground glass opacity (GGO) only	18 (36.0)
GGO + crazy paving appearance	32 (64.0)
FVC % predicted	81.1 ± 16.6
DL_CO_ % predicted	69.5 ± 25.2
PaO_2_ mm Hg	79.1 ± 24.7
D(A-a)O_2_ mm Hg	28.1 ± 15.2
Hb g/dL	14.1 ± 2.3
LDH U/L	419.3 ± 180.7
CEA ng/mL	7.0 ± 7.8
Follow-up days (median [IQR])	677 (214–1588)

*IQR* interquartile range, *FVC* forced vital capacity, *DL*_*CO*_ diffusing capacity of the lung for carbon monoxide, *Hb* hemoglobin, *LDH* lactate dehydrogenase, *CEA* carcinoembryonic antigen

^a^Parametric data are shown as means (± standard deviation) and nonparametric data as medians and interquartile ranges (IQRs). Dichotomous or discontinuous variables are presented as numbers (%)

^b^Data on arterial blood gas analyses could not be obtained in 13 patients. The categories of score ranged from DSS 1 to DSS 5 as described previously^17^; DSS 1 = no symptoms and PaO_2_ ≥ 70 mmHg, DSS 2 = symptomatic and PaO_2_ ≥ 70 mmHg, DSS 3 = 60 mmHg ≤ PaO_2_ < 70 mmHg, DSS 4 = 50 mmHg ≤ PaO_2_ < 60 mmHg, DSS 5 = PaO_2_ < 50 mmHg

**Table 2:** Diagnostic and treatment methods of the patients diagnosed with PAP between 1993 and 2014 in Korea (*n* = 78)

Variables^a^	*N* = 78
Diagnostic method	
Surgical biopsy	35 (44.9)
TBB with BAL	65 (83.3)
Treatment modality	
No	39 (50.0)
Whole lung lavage	35 (44.9)
Segmental lavage	3 (3.8)
GM-CSF^b^	4 (5.1)
Survivor	75 (96.2)

*TBB* transbronchial biopsy, *BAL* bronchoalveolar lavage, *GM-CSF* granulocyte-macrophage colony-stimulating factor

^a^Dichotomous or discontinuous variables are presented as numbers (%)

^b^Among the patients who were treated with GM-CSF, 3 patients were also treated by whole lung lavage

When we compared clinical and laboratory features between patients who received lavage and those who did not, patients with lavage more frequently experienced symptoms than did those without (100% vs. 60%, *P* < 0.001) (Table 3). In particular, presence of dyspnea was associated with treatment (90% vs. 40%, *P* < 0.001). Also, patients with lavage had significantly lower FVC (%), DL_CO_ (%), and PaO_2_ levels and higher D(A-a)O_2_ level at initial manifestation (*P* = 0.005, *P* < 0.001, *P* = 0.006 and *P* = 0.036, respectively). Consequently, there was a meaningful difference in the distribution of DSS between patients who underwent lavage and those who did not (*P* = 0.001). Regarding type of exposed dusts, farming dust was significantly associated with treatment (*P* = 0.011).

**Table 3:** Comparison of the clinical and laboratory features between patients who received lavage (*n* = 40) and those who did not (*n* = 38)

Variables^a^	No Lavage (*n* = 40)	Lavage (*n* = 38)	*P* value
Age (median years [IQR])	46 (39–54)	49 (44–61)	0.089
Male	24 (60.0)	23 (60.5)	0.962
Smoking status (*n* = 73)			0.878
Never smoker	19 (50.0)	15 (42.9)	
Ex-smoker	9 (23.7)	9 (25.7)	
Current smoker	10 (26.3)	11 (31.4)	
Presence of symptoms	24 (60.0)	38 (100.0)	<0.001
Presence of dyspnea	16 (40.0)	34 (89.5)	<0.001
Presence of dust exposure (*n* = 71)	19 (48.7)	15 (46.9)	0.403
Type of exposed dust			
Farming	0 (0)	5 (33.3)	0.011
Metal	6 (31.6)	3 (20.0)	0.697
Stone or sand	6 (31.6)	1 (6.7)	0.104
Chemical or paint	4 (21.0)	2 (13.3)	0.672
Diesel	3 (15.8)	2 (13.3)	1.000
Textile	0 (0)	1 (6.7)	0.441
Wood	0 (0)	1 (6.7)	0.441
Disease severity score^b^ (*n* = 65)			0.001
1	9 (31.0)	0 (0)	
2	13 (44.8)	20 (55.6)	
3	7 (24.1)	9 (25.0)	
4	0 (0)	4 (11.1)	
5	0 (0)	3 (8.3)	
FVC % predicted	86.7 ± 15.8	75.5 ± 15.7	0.005
DL_CO_ % predicted	82.3 ± 20.7	57.5 ± 23.3	<0.001
PaO_2_ mm Hg	88.2 ± 30.8	71.7 ± 15.4	0.006
D(A-a)O_2_ mm Hg	23.6 ± 14.5	31.6 ± 15.1	0.036
LDH U/L	389.4 ± 171.9	450.6 ± 188.6	0.239
CEA ng/mL	4.2 ± 1.9	10.3 ± 10.8	0.760

*IQR* interquartile range, *NA* not applicable, *FVC* forced vital capacity, *DL*_*CO*_ diffusing capacity of the lung for carbon monoxide, *LDH* lactate dehydrogenase, *CEA* carcinoembryonic antigen

^a^Parametric data are shown as means (± standard deviation) and nonparametric data as medians and interquartile ranges (IQRs). Dichotomous or discontinuous variables are presented as numbers (%)

^b^Data on arterial blood gas analyses could not be obtained in 13 patients. The categories of score ranged from DSS 1 to DSS 5 as described previously^17^; DSS 1 = no symptoms and PaO_2_ ≥ 70 mmHg, DSS 2 = symptomatic and PaO_2_ ≥ 70 mmHg, DSS 3 = 60 mmHg ≤ PaO_2_ < 70 mmHg, DSS 4 = 50 mmHg ≤ PaO_2_ < 60 mmHg, DSS 5 = PaO_2_ < 50 mmHg

When we classified the total patients into two groups (patients with low DSS [DSS 1–2] vs. patients with high DSS [DSS 3–5]) according to whether the patients had hypoxemia or not in arterial blood gas analyses at the time of diagnosis (Table 4), smoking status differed significantly between the two groups (*P* = 0.023). The proportion of current smokers were significantly higher in patients with high DSS than in those with low DSS (50% vs. 18%, *P* = 0.008). Regarding type of exposed dust, farming dust was significantly associated with high DSS (45% vs. 0%, *P* = 0.004).

**Table 4:** Comparison of the clinical and laboratory features between patients with low DSS (*n* = 42) and those with high DSS (*n* = 23)

Variables^a^	Low DSS^b^ (*n* = 42)	High DSS (*n* = 23)	*P* value
Age (median years [IQR])	48 (41–59)	46 (44–59)	0.878
Male	24 (57.1)	16 (69.6)	0.325
Smoking status (*n* = 61)			0.023
Never smoker	18 (46.2)	9 (40.9)	0.692
Ex-smoker	14 (35.9)	2 (9.1)	0.018
Current smoker	7 (17.9)	11 (50.0)	0.008
Presence of symptoms	33 (78.6)	22 (95.7)	0.084
Presence of dyspnea	25 (59.5)	19 (82.6)	0.057
Presence of dust exposure (*n* = 59)	18 (47.4)	11 (52.4)	0.712
Type of exposed dust			
Farming	0 (0)	5 (45.4)	0.004
Metal	6 (33.3)	3 (27.3)	1.000
Stone or sand	4 (22.2)	0 (0)	0.268
Chemical or paint	4 (22.2)	1 (9.1)	0.622
Diesel	2 (11.1)	2 (18.2)	0.622
Textile	1 (5.6)	0 (0)	1.000
Wood	1 (5.6)	0 (0)	1.000
FVC % predicted	83.2 ± 15.5	73.3 ± 16.1	0.05
DL_CO_ % predicted	75.6 ± 23.9	51.0 ± 22.8	0.001
PaO_2_ mm Hg	88.8 ± 25.2	61.4 ± 9.5	<0.001
D(A-a)O_2_ mm Hg	20.7 ± 11.1	42.2 ± 11.7	<0.001
LDH U/L	433.9 ± 182.4	352.6 ± 157.3	0.251
CEA ng/mL	6.4 ± 8.7	9.6 ± 8.6	0.294
Treatment			0.116
No	21 (50.0)	7 (30.4)	0.128
Whole lung lavage	18 (42.9)	16 (69.6)	0.039
Segmental lavage	2 (4.8)	0 (0)	0.536
GM-CSF^c^	3 (7.1)	0 (0)	0.547

*DSS* disease severity score, *IQR* interquartile range, *NA* not applicable, *FVC* forced vital capacity, *DL*_*CO*_ diffusing capacity of the lung for carbon monoxide, *LDH* lactate dehydrogenase, *CEA* carcinoembryonic antigen

^a^Parametric data are shown as means (± standard deviation) and nonparametric data as medians and interquartile ranges (IQRs). Dichotomous or discontinuous variables are presented as numbers (%)

^b^Data on arterial blood gas analyses could not be obtained in 13 patients among the total 78 patients. The categories of score ranged from DSS 1 to DSS 5 as described previously^17^; DSS 1 = no symptoms and PaO_2_ ≥ 70 mmHg, DSS 2 = symptomatic and PaO_2_ ≥ 70 mmHg, DSS 3 = 60 mmHg ≤ PaO_2_ < 70 mmHg, DSS 4 = 50 mmHg ≤ PaO_2_ < 60 mmHg, DSS 5 = PaO_2_ < 50 mmHg

^c^Among the patients who were treated with GM-CSF, two patients were also treated by whole lung lavage

**Fig. 2:**
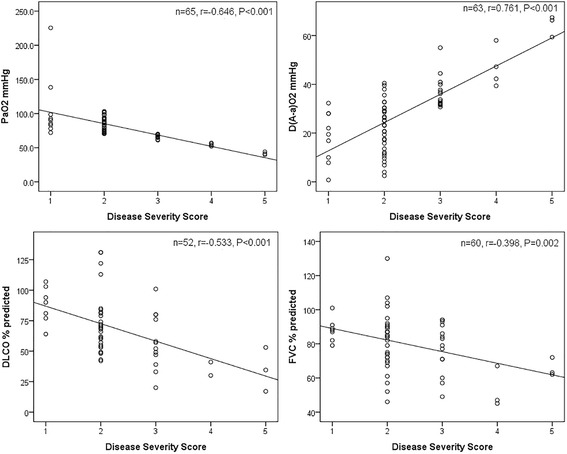
Correlations between DSS and D(A-a)O_2_, PaO_2_, DL_CO_(%), or FVC(%) in patients with PAP are shown with superimposed regression lines

Patients with high DSS had meaningfully lower FVC (%), DL_CO_ (%), and PaO_2_ levels, and higher D(A-a)O_2_ level than those with low DSS. Furthermore, DSS was positively correlated with D(A-a)O_2_ (*r* = 0.746, *P* < 0.001) and inversely with PaO_2_ (*r* = −0.646, *P* < 0.001), DL_CO_ (%) (*r* = −0.533, *P* < 0.001) and FVC (%) (*r* = −0.398, *P* = 0.002) (Fig. 2). However, DSS did not correlate with the known serum severity markers (DSS–LDH: *r* = −0.209; *P* = 0.222, DSS–CEA: *r =* 0.406; *P* = 0.215).

**Fig. 3:**
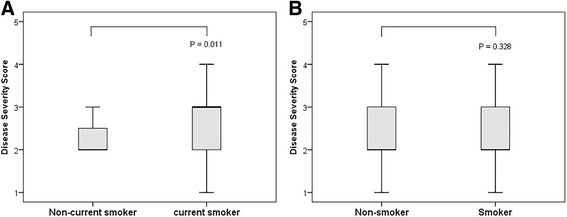
Box and whisker plots of the distribution of DSS between (**a**) non-current smokers (*n* = 43) and current smokers (*n* = 18) and between (**b**) non-smokers (*n* = 27) and smokers (*n* = 34). The box represents the interquartile range that contains 50% of the values, and the whiskers extending from the box represent the highest and lowest values. The thick line in the box represents the median value. There was a significant difference in DSS between non-current smokers and current smokers (A, *P* = 0.011), whereas no difference was found between non-smokers and smokers (B, *P* = 0.328)

Further analysis revealed that current smokers had significantly higher DSS (Fig. 3a) and serum CEA levels than non-current smokers (DSS: 2.8 ± 0.9 vs. 2.2 ± 0.9; *P* = 0.011, CEA: 8.9 ± 8.2 ng/mL vs. 5.4 ± 7.6 ng/mL; *P* = 0.031), whereas serum LDH level was not different between the two groups. However, when we categorized patients into smokers and non-smokers, no difference was found between the two groups with regard to DSS (Fig. 3b), serum CEA, or LDH level.

The total exposed dose of cigarette smoking (number of cigarette pack-years [PYs]) significantly correlated with indicators of PAP severity such as DSS (*r* = 0.567, *P* = 0.028), PaO_2_ (*r* = −0.597, *P* = 0.019), and D(A-a)O_2_ (*r* = 0.624, *P* = 0.017) in patients with both dust exposure and cigarette smoking history (*n* = 18). Such correlations were stronger in patients with both dust exposure and current active smoking at PAP diagnosis (*n* = 12; PY-PaO_2_: *r* = −0.627, *P* = 0.053; PY-D(A-a)O_2_: *r* = 0.724, *P* = 0.028).

## Discussion

Our present study investigated clinical characteristics of Korean PAP patients over 20 years, and examined the potential risk factors of PAP with a focus on smoking and dust exposure.

We classified the total patients into two groups according to whether or not the patients were treated with lavage and analyzed the differences. Treated patients presented symptoms more frequently and had significantly lower PaO_2_ and DL_CO_ (%) with higher D(A-a)O_2_ at the onset of disease than patients who were not treated. The presence of symptoms correlated with increased hypoxia and decreased diffusing capacity. Correspondingly, the distribution of DSS differed significantly between the two groups. Based on these results, we categorized all patients according to the level of DSS and discovered that current smoking is significantly associated with PAP severity. In support of this finding, we demonstrated that CEA levels, a biomarker for PAP severity, were higher in current smokers compared to non-current smokers. However, when we classified the patients into smokers and non-smokers, we could not find such a difference. Regarding type of exposed dust, farming dust was associated with more severe form of PAP.

Despite a few preexisting well-designed clinical studies, no other studies have demonstrated a relationship between cigarette smoking and PAP. Seymour and colleagues reported in their meta-analysis that 72% of PAP patients were smokers with male predominance, but no differences were found regarding symptoms, serum LDH level, PaO_2_, or D(A-a)O_2_ between smokers and non-smokers [5]. Also, one study from Germany reported that, among 70 PAP patients over a 30-year period, 79% were smokers [12], contrasting with 56% of 248 patients in a Japanese national study [10]. Although the proportion of smokers varies, all the above-mentioned studies have analyzed the difference between smokers and non-smokers and could not prove an association between smoking and PAP. However, our study revealed that active smoking at the onset of disease but not past smoking is associated with the severity of PAP, although it is not clear that cigarette smoking itself initiates or induces PAP. That might explain the study result of Bonella et al. in which active current smokers required a higher number of whole lung lavages for achieving remission than did non-smokers [12].

Then, what is the possible mechanism by which cigarette smoking affects the severity of PAP? First of all, it is notable that some similarities exist between PAP and other antibody-mediated systemic diseases with pulmonary manifestations such as Goodpasture’s syndrome. A causative relationship between active smoking and Goodpasture’s syndrome has been proposed in several previous studies based on the fact that a high proportion of patients with lung hemorrhage were active smokers. They suggested the possibility that cigarette smoke could increase the permeability of lung capillaries to allow preformed circulating antibodies to reach the alveolar basement membrane (ABM), or that smoking and other inhaled toxins could alternatively change antigenic determinants in the ABM, inducing formation of anti-ABM antibodies or enabling them to cross-react with anti-glomerular basement membrane (anti-GBM) antibodies [26, 27]. Moreover, similar to the former mechanism, some preclinical studies have shown that a high concentration of inspired oxygen or hydrocarbon fumes can induce permeability changes in lung capillaries [28–30].

It has also recently been demonstrated that cigarette smoke not only alters epithelial barrier functions, but also directly induces endothelial cell barrier disruption via oxidative stress-dependent and ceramide-mediated cytoskeletal changes in a dose- and time-dependent manner [31]. While breaches of the epithelial barrier might induce wound-repair inflammatory responses, disruption of the endothelial barrier can directly increase access of circulating proteins, plasma, and inflammatory cells to the interstitium and alveolar spaces [31–34]. Therefore, by inducing direct changes in the permeability of the lung capillaries, cigarette smoking can presumably enable preformed antibodies (Abs) like anti-GM-CSF Abs to leak out through the lung capillaries to reach alveolar spaces, causing iPAP. Due to the dose-dependent relationship between cigarette smoke and endothelial layer damage, we can speculate that PAP severity would change according to the amount of smoking. In consonance with this, our present study showed that significant correlations were observed between the total exposed dose of cigarette smoking and indicators of PAP severity in patients with both dust exposure and cigarette smoking history.

Prior studies of Lin et al. also revealed that, whereas the level of serum anti-GM-CSF Abs did not correlate with severity of iPAP, the level of anti-GM-CSF Abs in BAL fluid directly correlated with disease severity markers such as serum LDH, PaO_2_, D(A-a)O_2_ and DL_CO_, further predicting the need for subsequent therapeutic lavage, which implies the interrelation between degree of alveolar capillary damage and severity of iPAP [19, 21].

Meanwhile, serum LDH, KL-6, and CEA are well-established biomarkers related to severity of PAP which reflect alveolar epithelial cell damage to a certain degree [18, 22–25]. In the present study, although mean serum CEA level was elevated in both current and non-current smokers, currently smoking patients had significantly higher serum CEA level than non-current smokers (*P* = 0.031). However, there was no difference between smokers and non-smokers, similar to the previous result of Fujishima et al. [22], supporting that active smoking at the onset of PAP but not past smoking is associated with more severe disease manifestations. In addition, considering that serum CEA level is associated with smoking in a dose-dependent manner, however, after cessation of smoking, elevated CEA level decreases to the range of non-smokers [35, 36], the well-established relationship between serum CEA level and iPAP severity should be reappraised in terms of that between current smoking and severity of iPAP.

Regarding type of dust exposure, farming dust was associated with more severe form of PAP in our present study, although other types of exposed dusts revealed not to be associated with PAP severity, presumably due to the small number of patients. In possible relation to this, some preclinical studies have shown that the degree of lung injury differs according to the type and degree of dust exposure [17, 37–40]. Although several case studies showing the effects of dust exposure on PAP have been reported to date [41–45], extensive clinical studies enrolling a large number of PAP patients are needed to confirm this finding.

Several limitations of this study should be noted. First, it was not possible to measure the level of serum anti-GM-CSF Abs, although idiopathic PAP in this study could be assumed to be autoimmune PAP. Second, this was a retrospective observational study that included a small number of PAP cases with information on dust exposure obtained from limited occupational history. However, considering the rare incidence of PAP, this study is notable because, as a multi-center study conducted in one country, it focused on the possible risk factors of PAP and demonstrated the effects of cigarette smoking on PAP severity for the first time. Further studies are needed to determine whether cigarette smoking can directly induce PAP and to demonstrate the effects of dust exposure on PAP severity in a large PAP cohort.

## Conclusion

In conclusion, a considerable proportion of PAP patients had a history of cigarette smoking and/or dust exposure, suggestive of their possible roles in the development of PAP. Active cigarette smoking at the onset of PAP is associated with the severity of PAP.

## Abbreviations

Ab: Antibody
ABM: Alveolar basement membrane
AM: Alveolar macrophage
CEA: Carcinoembryonic antigen
DL_CO_: Diffusing capacity of the lung for carbon monoxide
DSS: Disease severity score
FVC: Forced vital capacity
GM-CSF: Granulocyte-macrophage colony-stimulating factor
iPAP: Idiopathic pulmonary alveolar proteinosis
LDH: Lactate dehydrogenase
PAP: Pulmonary alveolar proteinosis
PFT: Pulmonary function test
WLL: Whole lung lavage
